# Office Notes

**Published:** 1891-05

**Authors:** William H. Rollins


### OFFICE NOTES.[1]

BY DR. WILLIAM H. ROLLINS.

*Clean Instruments.*—Dental instruments should be sterilized to prevent the spread of Riggs's disease, if for no other reason.

What is the best means of rendering them aseptic? We use so many instruments in comparison with the surgeon that the matter is one taking considerable time, and unless a cheap and easy method is used the procedure will be neglected. After trying several ways used in general surgery I have adopted baking as the only practical one. A good oven which has a non-conducting covering is essential, and any of the gas cooking-stoves made by the Goodwin Gas Stove and Meter Company, answer the purpose perfectly. I

[1] Read before the Odontological Society of New York, February 17, 1891.

recommend the No. 7 as having sufficient capacity for the purpose. By testing the heat of the oven the first time, it is always easy to get the same heat again, without the use of a thermometer, by observing the size of the flame. A more accurate way would be to use one of the regulating devices in common use, but I have not yet done this. The difficulty of this method is that the wood of the handles is injured by repeated exposures to a temperature of 300° F., and this is as low a temperature as should be used. As soon as aluminum became cheap enough, Codman & Shurtleff began to use it extensively for handles to their surgical instruments. When properly plated, such handles are satisfactory, and can easily be sterilized. Aluminum is so light that it is well adapted for handles of dental instruments. It is too soft to hold the thread of socket instruments, and on this account the sockets should be made of steel and screwed into the handles. I send for inspection one of these handles which Codman & Shurtleff made for me.

Mouth-mirrors and syringes are difficult to keep aseptic. The former deteriorate rapidly. I have used on an average one a month. During the time I have tried to keep clean instruments, and, on applying to the S. S. White Company, to see if anything could be done to make them more durable, I got no satisfaction, being told that a mouth-mirror should never be washed nor heated,—simple wiping being all that was allowable. Imagine using an instrument in a patient's mouth that has been simply wiped! In syringes the leather soon gives out, and to avoid this I have my syringe pistons made of brass, with hard rubber or other split rings like those in a steam engine. There should be four rings to insure a sufficiently tight piston. Such a piston is not as tight as a new leather one, but the fact that one of mine has been in constant daily use for three years shows that, for the ordinary washing out of the cavities in teeth, it is good enough. The rubber itself does not seem to wear, the wear coming on the metal of the syringe. These syringes can be had of Codman & Shurtleff.

*Electric Action from Amalgam.*—Seven months ago I put a gold crown on an inferior molar. The back of the bicuspid and the adjoining tooth had both been some years previously filled with amalgam. As the patient did not return to town till late, I did not see her again until December, when she came in for a moment complaining that the cap cut her tongue. As she was ill, I did not examine her teeth at this time, simply polishing the surface of the crown next the tongue, though I could not find any special roughness.

About two weeks ago the patient, who was very ill, sent for me. The entire membrane of the mouth was congested; there was an increased flow of saliva; and on the tongue opposite the gold cap there was an ulcer. She was unable to take any nourishment except milk, on account of the soreness of the mouth and tongue, and was suffering such pain in the ear that sleep without medicine was impossible. I gave her a soothing wash, and soon thereafter examined the teeth carefully.

There was a silver-colored spot on the gold crown where it came near the amalgam. In several short sittings I removed the gold cap and the amalgam fillings. In a week the flow of saliva was diminished, the mucous membranes of the mouth were normal in appearance, and the ulcer had healed. The pain in the ear has not yet entirely gone.

Whatever the explanation may be, the case shows that, under certain circumstances, the use of amalgam is not desirable. Certain abnormal general conditions of the system may make a patient more susceptible to the presence of these fillings, especially where there are other metals in the teeth.

I hope to examine the silver-colored spot on the gold cap, and shall then be able to express an opinion. I have delayed the examination, as I wished to show the cap as it was in the mouth.

I tested the parotid saliva twice in this case before removing the fillings and found it acid. The patient has been, and still is, suffering severely from gout.

I send on the gold cap for the inspection of the members of the Odontological Society.

*Acid-Washing of Amalgam.*—Most of the makers of amalgam alloy state that alloys should not be washed, and many dentists follow their directions. Then there are others who use either alcohol or bicarbonate of soda, and wash with these.

A year ago I suggested to Dr. Russell, whose alloy I use, that he should bring my method of acid-washing to the attention of dentists, but as I have not seen the method in print, I wish to bring it before the Society, as it makes amalgamation easy and gives a clean material, which is also stronger than an unwashed amalgam.

I keep, in a convenient place, a solution of sulphuric acid in water, one part in fifty. A little of this is poured into a small glass mortar, enough of the alloy put in and worked for a moment with the pestle; when the alloy is perfectly clean, shake in the required amount of mercury and rub together. Amalgamation is almost immediate. Pour off the weak acid, and wash in a few changes of water, then dry and use. Sulphuric acid is a convenient acid for the purpose, as it can be poured into the waste pipe without danger, and it forms soluble salts with the metals of the alloy.

*Warm Instruments.*—No dental instrument should be used at a lower temperature than the blood. This may seem a small matter, but constant daily use has shown me that it is a source of comfort to patients. It is necessary that the means used to warm the instruments should be simple and automatic, otherwise it will not be done. I have a closed copper vessel with flat top, measuring twenty by thirty inches, by one inch in depth. In one corner of the upper surface is a small upright pipe like the neck of a bottle, which is closed with a cork. This flat vessel covers the top of my operating case, one end projecting enough to allow a gas-burner to be placed under it without risk of burning the wood-work of the case.

Before use, the vessel is half-filled with water and the cork put in. A simple automatic regulator turns down the gas when the temperature has reached the proper point and maintains it there all day. I select the instruments to be used, and put them on the pan, where they warm in a few minutes. In addition to the instruments, six small glasses of water are kept on the closed pan; in one of these is a small chemical thermometer, which shows that the temperature is right; in another I keep the syringe for washing out the decayed cavities. The other glasses are for the use of patients in rinsing the mouth. If for no other purpose, it would be worth while to have such an apparatus to furnish a supply of properly warmed water without any care or attention. There is, moreover, not the least doubt that properly warmed instruments hurt less than when used at the temperature of the operating-room. My warming vessel was made by Peter Gray, 12 Marshall Street, Boston.

During the twelve years that I have used warm instruments I have converted one dentist to my way of thinking; but the importance of the matter should not be judged by the number of converts.

*New Preparations for Dental Use.*—I have prepared an arseniate of cocaine which has some good points in treatment of the dental pulp. Applied to a pulp which is to be destroyed, it accomplishes this without much pain. In a weak solution it diminishes the sensibility of dentine.[1] I have a few cases under observation on which I

---

[1] This statement cannot be published without a protest. The use of arsenious acid and cocaine in combination to reduce the sensitiveness of dentine can have but one result, the destruction of the pulp. This question was settled years ago, and it is remarkable that it should be again revived.—Ed.

hope to report in a year or two, but if it could be tested on sensitive dentine in hospital practice the cases would be so much more numerous that definite information might be had in a shorter time. In private practice it is not desirable to try it as an obtunder except in certain teeth, like bicuspids or others that are to be removed for regulating, for it might endanger the vitality of the dental pulp. I have asked Theodore Metcalf & Co., Boston, to prepare some of the arseniate in case any dentist should wish to experiment with it.

